# Epstein–Barr Virus Silences GSDME and Pyroptosis in Gastric Cancer

**DOI:** 10.3390/microorganisms13122704

**Published:** 2025-11-27

**Authors:** Min-Hyeok Lee, Ju Yeon Lee, Jun Yeob Kim, Yea Rim An, Suk Kyeong Lee

**Affiliations:** 1 Department of Medical Life Sciences, College of Medicine, The Catholic University of Korea, Seoul 06591, Republic of Korea; aksgur0627@catholic.ac.kr (M.-H.L.); jjing1004@catholic.ac.kr (J.Y.L.); kjygod1@catholic.ac.kr (J.Y.K.);; 2 Department of Medical Sciences, College of Medicine, The Catholic University of Korea, Seoul 06591, Republic of Korea

**Keywords:** Epstein–Barr virus, gastric cancer, *GSDME*, pyroptosis, caspase-3, DNA methylation, paclitaxel, epigenetic regulation

## Abstract

Epstein–Barr virus (EBV)-associated gastric carcinoma (EBVaGC) represents a distinct molecular subtype characterized by extensive DNA methylation and altered cell death signaling. This study investigated the regulation and function of gasdermin E (GSDME), a key mediator of pyroptosis, in EBVaGC. Transcriptomic analysis of The Cancer Genome Atlas (TCGA) data revealed that *GSDME* expression was selectively suppressed in EBV-positive gastric cancer, while other gasdermin family members were upregulated. Validation in multiple cell lines confirmed that EBV infection markedly reduced *GSDME* expression through promoter hypermethylation, which was reversed by treatment with the DNA methyltransferase inhibitor 5-azacytidine. EBV-positive cells exhibited enhanced caspase-3 activation and increased GSDME cleavage upon paclitaxel (PTX) exposure, leading to elevated lactate dehydrogenase (LDH) release and pyroptotic morphology. Overexpression of *GSDME* amplified, whereas siRNA-mediated knockdown or caspase-3 inhibition suppressed, PTX-induced pyroptosis without significantly altering overall cell viability. These findings demonstrate that EBV-induced epigenetic silencing of *GSDME* contributes to the modulation of chemotherapy-induced cell death, and that GSDME acts as a critical effector converting apoptosis to pyroptosis through caspase-3 activation. Collectively, our results reveal a novel link between EBV-driven DNA methylation and pyroptotic cell death, suggesting that restoration of *GSDME* expression may enhance therapeutic responses in EBV-associated gastric cancer.

## 1. Introduction

Epstein–Barr virus (EBV) is a ubiquitous γ-herpesvirus that infects more than 90% of the global population [1] and is etiologically linked to various malignancies, including nasopharyngeal carcinoma, Hodgkin lymphoma, and EBV-associated gastric carcinoma (EBVaGC) [2,3,4]. During latent infection, EBV restricts the expression of viral genes through DNA methylation, enabling immune evasion and lifelong persistence within the host [5,6,7]. Concurrently, EBV promotes the hypermethylation of host tumor suppressor genes by upregulating DNA methyltransferases, leading to a CpG island methylator phenotype that contributes to gastric carcinogenesis [4,8]. In addition, EBV latent proteins and viral microRNAs regulate cell proliferation, apoptosis, and necroptosis, creating a cellular environment conducive to oncogenic transformation and tumor progression [9,10,11]. While the effects of EBV on apoptosis, necroptosis, and autophagy have been extensively studied, its influence on pyroptosis, an inflammatory form of programmed cell death, remains largely unexplored.

Pyroptosis is a lytic and pro-inflammatory type of programmed cell death mediated by the gasdermin protein family [12,13]. Upon activation, specific caspases cleave gasdermins, releasing their N-terminal fragments, which oligomerize to form plasma membrane pores, leading to cell swelling, rupture, and release of inflammatory mediators such as interleukin (IL)-1β, IL-18, HMGB1, and ATP [14]. The canonical pathway involves cleavage of gasdermin D (GSDMD) by inflammatory caspases (caspase-1, -4, -5, and -11) during inflammasome activation [15,16]. More recently, gasdermin E (GSDME, also known as DFNA5) has been identified as a substrate of caspase-3, a key apoptosis executioner, thereby serving as a molecular switch between apoptosis and pyroptosis [17].

Apoptosis maintains plasma membrane integrity and is generally immunologically silent, whereas pyroptosis triggers membrane rupture and the release of damage-associated molecular patterns (DAMPs), leading to immune cell activation and potential enhancement of antitumor immunity [18,19,20]. Several chemotherapeutic agents, including cisplatin, doxorubicin, and paclitaxel (PTX), can induce caspase-3-dependent cleavage of GSDME, resulting in pyroptotic cell death [21,22,23]. However, *GSDME* expression is frequently silenced by promoter hypermethylation in multiple cancers, including gastric cancer, which prevents pyroptosis and favors non-inflammatory apoptosis [22,24,25].

Gastric cancer remains a major global health burden, ranking fifth in incidence and mortality worldwide according to the GLOBOCAN 2022 report by the International Agency for Research on Cancer (IARC) [26]. The Cancer Genome Atlas (TCGA) project has classified gastric cancer into four molecular subtypes, among which EBVaGC accounts for approximately 10% of all cases [27]. This subtype is distinguished by extensive immune cell infiltration and unique viral gene expression patterns [27,28].

EBVaGC is also characterized by extensive epigenetic silencing of tumor suppressor genes through promoter hypermethylation, suggesting that *GSDME* may also be a target of EBV-mediated epigenetic repression [28,29]. Indeed, previous studies have reported *GSDME* promoter methylation in up to 50% of gastric cancers, with a subset showing a significant association with EBV infection [30]. However, it remains unclear whether EBV infection directly induces *GSDME* hypermethylation and how this alteration affects the balance between apoptosis and pyroptosis, particularly under chemotherapeutic stress.

In this study, we investigated the relationship between EBV infection, *GSDME* expression, and promoter methylation in gastric cancer using TCGA datasets and a panel of EBV-negative, artificially EBV-infected, and naturally EBV-infected gastric cancer cell lines. Furthermore, we explored how EBV infection modulates chemotherapy-induced pyroptosis via the caspase-3-GSDME axis. Our findings provide new insight into the epigenetic mechanisms by which EBV regulates cell death pathways and suggest that restoration of *GSDME* expression may enhance therapeutic responsiveness in EBVaGC.

## 2. Materials and Methods

### 2.1. Cell Culture and Drug Treatment

AGS (gastric adenocarcinoma), SNU216 (gastric adenocarcinoma), MKN45 (gastric adenocarcinoma), and SNU719 (naturally EBV-infected gastric adenocarcinoma) [31] cell lines were purchased from the Korean Cell Line Bank (Seoul, Republic of Korea). MKN1 (gastric adenosquamous carcinoma) and MKN1-EBV cell lines were kindly provided by Prof. Hyojeung Kang (Institute of Pharmaceutical Science, Kyungpook National University, Daegu, Republic of Korea). MKN1-EBV cells were established using a recombinant Akata EBV bacmid originally developed by Dr. Teru Kanda [32] and subsequently used to infect MKN1 cells [33]. The AGS-EBV cell line (recombinant Akata EBV-infected AGS) [34] was provided by Dr. Kenzo Takada (Institute for Genetic Medicine, Hokkaido University, Japan), and the YCCEL1 cell line (naturally EBV-infected gastric adenocarcinoma) [35] was obtained from Professor Sun Young Rha (Yonsei University College of Medicine, Seoul, Republic of Korea).

AGS, AGS-EBV, MKN1, MKN1-EBV, SNU216, MKN45 and SNU719 cells were cultured in Roswell Park Memorial Institute (RPMI) 1640 medium (Gibco, Grand Island, NY, USA). YCCEL1 cells were maintained in Eagle’s Minimal Essential Medium (EMEM; Lonza Benelux BV, Breda, The Netherlands). All media were supplemented with 10% fetal bovine serum (FBS, Corning, NY, USA), 100 μg/mL streptomycin, and 0.25 μg/mL amphotericin B. To maintain EBV-infected cells, AGS-EBV cells were cultured in medium containing 400 μg/mL G418 (Invivogen, San Diego, CA, USA), and MKN1-EBV cells were cultured in medium containing 200 μg/mL Hygromycin B (Invitrogen, Waltham, MA, USA). All cells were incubated at 37 °C in a humidified atmosphere with 5% CO_2_.

For experiments, cells were seeded at a density of 4 × 10^5^ cells per 60-mm dish and incubated for 24 h prior to treatment. For viability and cell death assays (MTT and LDH), cells were seeded at 4 × 10^3^ cells per well in 96-well plates. In experiments involving gene overexpression or siRNA-mediated knockdown, transfection was performed 4 h before drug treatment. For caspase inhibition, cells were pre-treated with Z-DEVD-FMK for 1 h prior to drug exposure. In other experiments, cells were directly treated with PTX, 5-fluorouracil (5-FU), or cisplatin for 48 h unless otherwise specified.

### 2.2. GSDME Overexpression

The human *GSDME* coding sequence (NM_001127453) was PCR-amplified from cDNA generated using AGS cell mRNA. The PCR product contained XhoI and EcoRI consensus restriction sites at each end (Table 1) and was inserted into the pcDNA3.1 (+) vector (Invitrogen), digested with the enzymes to generate the *GSDME* overexpression plasmid (pcGSDME). AGS and AGS-EBV cells were transfected with pcGSDME or empty vector (pcDNA3.1) (250 ng/mL) using Lipofectamine 2000 (Invitrogen) according to the manufacturer’s protocol. Twenty-four hours after transfection, cells were treated with PTX for 48 h.

### 2.3. GSDME Knockdown

MKN1 and MKN1-EBV cells were transfected with 20 nM si*GSDME* (GenePharma, Shanghai, China) using Lipofectamine RNAiMAX (Invitrogen) following the manufacturer’s instructions. The siRNA sequences (5′-3′) were as follows: negative control siRNA (siNC): UUCUCCGAACGUGUCACGUTT; si*GSDME* #1: GUGGCUUCGAGAACAAGAATT; si*GSDME* #2: GGCGGUCCUAUUUGAUGAUTT; si*GSDME* #3: GCAGCAA-GCAGCUGUUUAUTT; si*GSDME* #4: GCUGCAAACUCCAGAUCAUTT.

### 2.4. Total RNA Purification and Quantitative Real-Time PCR (qRT-PCR)

Total RNA was extracted from gastric cancer cell lines using RNAiso Plus (Takara, Shiga, Japan) according to the manufacturer’s protocol. cDNA was synthesized from 3 μg of total RNA using oligo (dT) primers and M-MLV reverse transcriptase (Enzynomics, Daejeon, Republic of Korea). qRT-PCR was performed using a SYBR Green qPCR premix (RT500S, Enzynomics) on a CFX96 real-time PCR system (Bio-Rad, Hercules, CA, USA). Primer sequences are listed in Table 1. Relative expression levels were calculated using ΔCq values and normalized to *GAPDH* expression.

### 2.5. Western Blot Analysis

Cells were lysed and mixed with 5× SDS loading buffer (Dynebio, Seoul, Republic of Korea), then heated at 95 °C for 5 min. Protein samples were separated by 12% or 15% SDS-PAGE and transferred onto polyvinylidene fluoride (PVDF) membranes (Millipore, Billerica, MA, USA). Membranes were blocked with 5% skim milk or 5% bovine serum albumin (BSA) for 30 min and incubated overnight at 4 °C with the following antibodies: anti-GSDME (1:1000, Abcam, Cambridge, UK; ab215191), anti-GSDMD (1:1000, Abcam; ab210070), anti-cleaved caspase-3 (1:1000, Cell Signaling Technology, Danvers, MA, USA; 9661), and anti-β-actin (1:1000, Cell Signaling Technology; 4070). After washing, membranes were incubated with HRP-conjugated anti-rabbit secondary antibody (1:2000, Cell Signaling Technology; 3900) for 1 h. Protein bands were visualized using an enhanced chemiluminescence detection system (Amersham Biosciences, Piscataway, NJ, USA) and exposed to X-ray film (Agfa, Mortsel, Belgium).

### 2.6. Demethylation by 5-Aza-2′ Deoxycytidine (5-AZA)

Gastric cancer cell lines were seeded in 60-mm culture dishes and, after 24 h, treated with 5-AZA (Sigma-Aldrich, St. Louis, MO, USA) for 48 h. The treated cells were harvested for analysis of *GSDME* expression and promoter methylation status.

### 2.7. Methylation-Specific PCR (MSP-PCR)

Genomic DNA was extracted using a DNA Mini Kit (Qiagen, Hilden, Germany; 51304). Bisulfite conversion and purification were performed using the EZ DNA Methylation Lightning Kit (Zymo Research, Irvine, CA, USA; D5030) following the manufacturer’s instructions. MSP was conducted using the AccuPower Epigene Methylation-Specific PCR Premix (Bioneer, Daejeon, Republic of Korea; K2410). Methylated (M) and unmethylated (U) DNA-specific primers were designed using MethPrimer (https://methprimer.com/; accessed on 2 December 2024) to target CpG islands within the *GSDME* promoter region. All MSP primers were designed to amplify the bisulfite-converted antisense DNA strand. Primer sequences are listed in Table 1.

### 2.8. Quantification of Pyroptosis

Flow cytometry-based cell death analysis was not feasible because EBV-positive cells exhibited strong baseline GFP fluorescence and a marked increase in green fluorescence after anticancer drug treatment, which interfered with Annexin V and PI signal detection. Therefore, pyroptotic cell death was quantified by a fluorescence microscopy–based morphological assessment. Cells (4 × 10^5^) were seeded in 60-mm culture dishes and incubated for 24 h, followed by treatment with the indicated compounds for an additional 48 h.

For each experimental condition, six random, non-overlapping microscopic fields showing approximately 50% confluency were captured at 200× magnification by two independent investigators in a blinded manner. Each field contained approximately 30–40 cells, and more than 200 cells were analyzed per sample. Cells exhibiting characteristic pyroptotic features, including cell swelling and bubble-like plasma membrane protrusions, were scored as pyroptotic, whereas cells showing membrane blebbing without ballooning morphology were classified as other forms of cell death. Pyroptosis was expressed as the percentage of pyroptotic cells among total dead cells, and mean values from three independent experiments were used for statistical comparisons.

### 2.9. Propidium Iodide (PI) Uptake Assay

To assess plasma membrane permeabilization following PTX treatment, a PI uptake assay was performed [36]. MKN1 and MKN1-EBV cells were seeded in an 8-well chambered coverglass (Thermo Fisher Scientific, Waltham, MA, USA). After 24 h, cells were transfected with siNC or si*GSDME* #2 and subsequently treated with PTX for 48 h. PI (2.5 μg/mL) was added directly to the culture medium for 1 h before imaging. For each condition, one randomly selected field per well was imaged from three independent wells (*n* = 3) using fluorescence microscopy. Total cell numbers were determined from bright-field images, and PI-positive cells were identified from the corresponding fluorescence images. Small fluorescent debris without a discernible cell body was excluded from analysis. When fragmented nuclear PI staining was observed, fragments belonging to the same cell were counted as a single PI-positive cell. To minimize observer bias, two independent investigators quantified PI-positive cells in a blinded manner. The percentage of PI-positive cells was calculated as follows: PI uptake (%) = (Number of PI-positive cells/Total number of cells per field) × 100.

### 2.10. Cytotoxicity Assay

To assess PTX-induced membrane damage, LDH release was measured as an indicator of pyroptotic cell death in the culture supernatant [37]. Cells (4000 cells/well) were seeded in 96-well plates and cultured for 48 h in phenol red-free RPMI medium (Gibco, 11835030) containing 0, 10, or 30 nM PTX. LDH release was quantified using the CytoTox 96^®^ Non-Radioactive Cytotoxicity Assay Kit (Promega, Madison, WI, USA, G1780) according to the manufacturer’s protocol.

### 2.11. MTT Assay (Cell Viability Assay)

Cell viability following PTX treatment was determined by MTT assay (Amresco, Shanghai, China). Cells (4000 cells/well) were seeded in 96-well plates and subjected to plasmid or siRNA transfection, or caspase-3 inhibitor pretreatment, followed by PTX treatment for 48 h. After treatment, the medium was replaced with 100 μL serum-free medium containing 10 μL MTT solution (5 mg/mL) and incubated for 2 h at 37 °C. The supernatant was then removed, and 100 μL dimethyl sulfoxide (DMSO) (Sigma-Aldrich) was added to dissolve the formazan crystals. Absorbance at 590 nm was measured using a SoftMax microplate reader (Molecular Devices, Sunnyvale, CA, USA).

### 2.12. Statistical Analyses

Data were analyzed using GraphPad Prism version 8.4.2 (GraphPad, San Diego, CA, USA). Two-way analysis of variance (ANOVA) was used for MTT and LDH assay data involving two independent variables, whereas comparisons between two groups were performed using Student’s *t*-test. Results are expressed as the mean ± standard deviation (SD) from three independent experiments.

## 3. Results

### 3.1. EBV Infection Selectively Suppresses GSDME Expression

**Figure 1:**
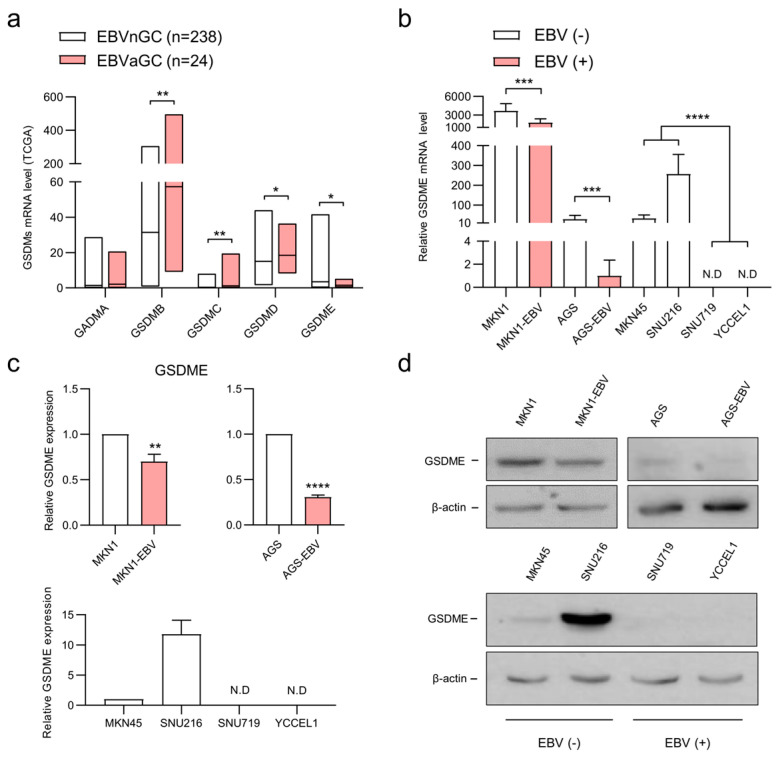
EBV infection suppresses *GSDME* expression. (**a**) RNA sequencing data from TCGA show the expression level of gasdermin family genes in EBVnGC (*n* = 238) and EBVaGC (*n* = 24). (**b**) mRNA expression of *GSDME* was analyzed via qRT-PCR in four EBV-negative (MKN1, AGS, MKN45 and SNU216) and four EBV-positive (MKN1-EBV, AGS-EBV, SNU719 and YCCEL1) gastric cancer cell lines. *GSDME* expression levels were normalized to *GAPDH* and calculated relative to AGS-EBV cells. (**c**) GSDME protein expression quantified by densitometric analysis of Western blots from three independent experiments. Band intensities were normalized to β-actin and expressed as fold changes relative to control cells (MKN1 and AGS for the upper panel; MKN45 for the lower panel). Data are presented as mean ± SD. (**d**) Representative Western blot images corresponding to the quantification shown in (**c**). β-actin was used as a loading control. Statistical significance is indicated as * *p* < 0.05, ** *p* < 0.01, *** *p* < 0.001, **** *p* < 0.0001. N.D, Not detected.

To investigate the impact of EBV infection on gasdermin family gene expression in gastric cancer, RNA sequencing data from TCGA were analyzed. Compared with EBV-negative gastric cancer (EBVnGC), EBVaGC exhibited increased expression of *GSDMB*, *GSDMC*, and *GSDMD*, whereas *GSDME* expression was significantly reduced (Figure 1a). The expression of *GSDMA* showed no significant difference between the two groups. To validate these findings at the cellular level, qRT-PCR and Western blot analyses were performed using EBV-negative (AGS, MKN1, SNU216, and MKN45), naturally EBV-infected (SNU719 and YCCEL1), and artificially EBV-infected (AGS-EBV and MKN1-EBV) gastric cancer cell lines (Figure 1b–d). Both mRNA and protein analyses revealed consistently reduced *GSDME* expression in EBV-infected cells compared with their EBV-negative counterparts, with almost undetectable expression in SNU719 and YCCEL1. Collectively, these findings demonstrate that EBV infection selectively downregulates *GSDME* expression in gastric cancer.

Among the tested cell lines, MKN1 cells exhibited the highest basal *GSDME* expression, while AGS cells showed markedly reduced expression even before EBV infection. Accordingly, AGS/AGS-EBV cells were used for *GSDME* overexpression studies, and MKN1/MKN1-EBV cells were used for siRNA-mediated knockdown experiments.

### 3.2. Reduced GSDME Expression in Gastric Cancer Is Associated with Promoter Hypermethylation

**Figure 2:**
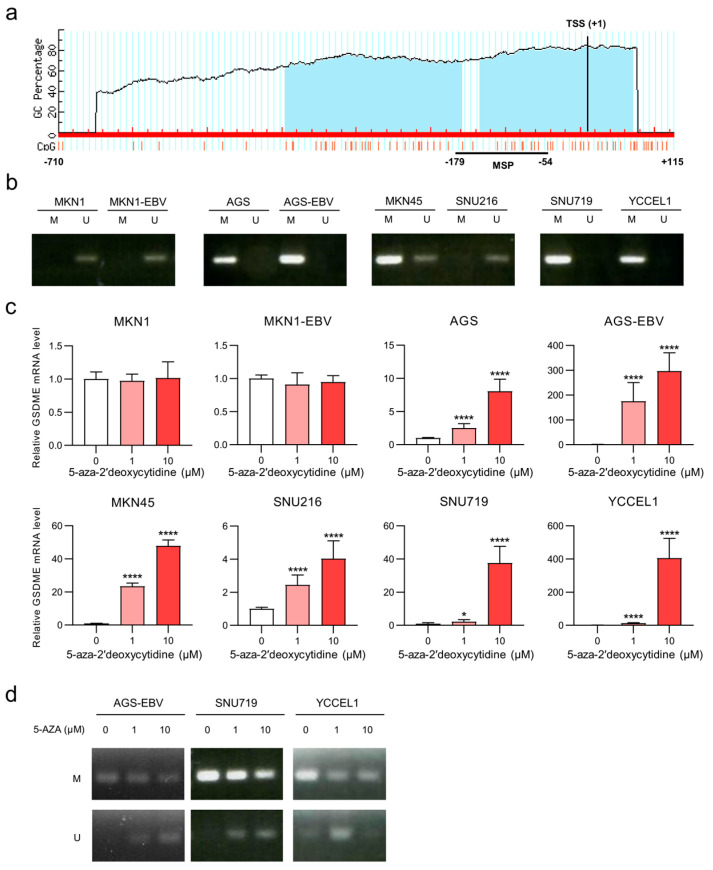
*GSDME* expression is suppressed by promoter hypermethylation. (**a**) Schematic representation of CpG islands predicted in the promoter region of the *GSDME* gene using MethPrimer. The blue region indicates CpG islands, and the black bar (−179 bp to −54 bp relative to the TSS) represents the MSP amplified region. Red vertical ticks represent CpG dinucleotide sites. (**b**) MSP analysis of *GSDME* in EBV-negative and EBV-positive gastric cancer cell lines. Unmethylated and methylated PCR products are labeled as ‘U’ and ‘M’, respectively. (**c**) Dose-dependent changes in *GSDME* mRNA expression after treatment with 0, 0.1, 1 and 10 μM 5-AZA in EBV-negative and EBV-positive cell lines. *GSDME* mRNA levels were normalized to untreated control cells. (**d**) MSP analysis showing demethylation of the *GSDME* promoter in SNU719, YCCEL1, and AGS-EBV cells after 48 h treatment with 5-AZA. Data are presented as mean ± SD (*n* = 3) and statistical significance is indicated as * *p* < 0.05, **** *p* < 0.0001.

To determine whether the reduction of *GSDME* expression is regulated epigenetically, we analyzed the methylation status of the promoter region encompassing approximately 900 bp around the transcription start site (TSS). MethPrimer analysis identified a CpG island within the *GSDME* promoter (Figure 2a). MSP revealed strong promoter methylation in AGS, AGS-EBV, YCCEL1, and SNU719 cells, which exhibited low *GSDME* expression. In contrast, no methylation was detected in MKN1, MKN1-EBV, or SNU216 cells, which expressed high *GSDME* levels, while MKN45 displayed a mixed methylation pattern consistent with intermediate expression (Figure 2b). Treatment with the DNA methyltransferase inhibitor 5-AZA induced a dose-dependent increase in *GSDME* mRNA levels in cell lines with low basal expression, whereas no significant change was observed in MKN1 or MKN1-EBV cells. Notably, SNU216, which expressed moderate basal levels, showed increased expression following 5-AZA treatment (Figure 2c). Consistently, MSP analysis confirmed demethylation of the *GSDME* promoter in AGS-EBV, SNU719, and YCCEL1 cells after 5-AZA treatment (Figure 2d).

These results indicate that promoter hypermethylation is the primary mechanism responsible for *GSDME* suppression in gastric cancer and that DNMT inhibition can restore *GSDME* expression by reversing CpG island methylation.

### 3.3. EBV Infection Augments Chemotherapy-Induced GSDME-Dependent Pyroptosis in Gastric Cancer Cells

**Figure 3:**
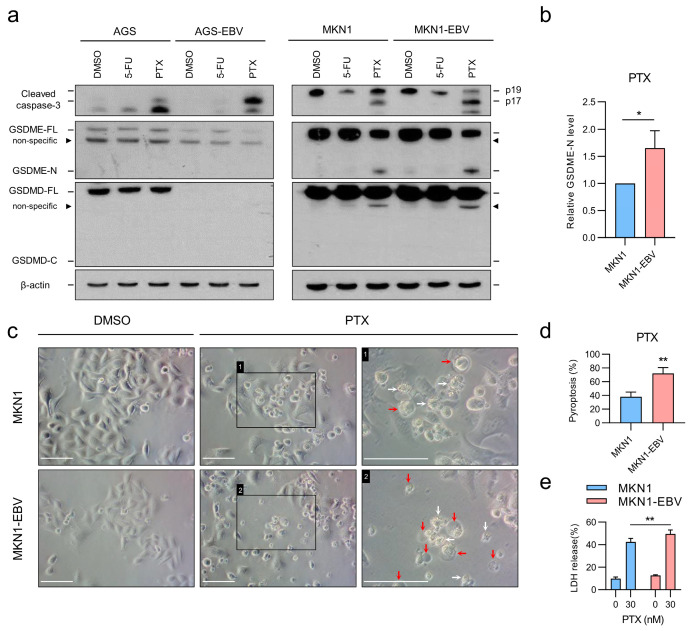
Effect of chemotherapeutic agents on caspase-3 and GSDME cleavage and pyroptosis in EBV-negative and EBV-positive cell lines. (**a**) EBV-negative and EBV-positive cell lines were treated with 10 μM 5-FU or 30 nM PTX for 48 h. Western blot analysis was performed for caspase-3 and gasdermin proteins. Black triangles indicate non-specific bands. β-actin was used as a loading control. (**b**) Densitometric quantification of N-terminal fragment of GSDME (GSDME-N) normalized to full-length GSDME (GSDME-FL), based on three independent experiments shown in (**a**). (**c**) Representative microscopic images of MKN1 and MKN1-EBV cells treated with PTX for 48 h. Red arrows indicate pyroptotic cells, and white arrows indicate non-pyroptotic cell death. Scale bar: white horizontal line, 100 µm. (**d**) Pyroptosis (%) was quantified by blinded, morphology-based analysis. Cells were treated for 48 h and imaged at ×200 magnification. Two independent investigators evaluated more than 200 cells per condition from randomly selected, non-overlapping fields. Cells exhibiting characteristic bubble-like plasma membrane protrusions were classified as pyroptotic, whereas dying cells lacking bubble formation or showing membrane blebbing were categorized as other forms of cell death. Data represent the mean percentages of pyroptotic cells from three independent biological experiments. (**e**) LDH release measured in the supernatant of MKN1 and MKN1-EBV cells after PTX treatment. Data are presented as mean ± SD (*n* = 3). Statistical significance is indicated as * *p* < 0.05, ** *p* < 0.01.

To assess whether EBV infection influences chemotherapy-induced cell death pathways, paired EBV-negative and EBV-positive cell lines (AGS/AGS-EBV, MKN1/MKN1-EBV) were treated with 5-FU or PTX. Western blot analysis demonstrated increased caspase-3 activation in EBV-infected cells compared with EBV-negative counterparts under both treatments. Cleaved GSDME (GSDME-N) was detected in MKN1 and MKN1-EBV cells, and its level was higher in MKN1-EBV. GSDME cleavage was barely detectable in AGS or AGS-EBV cells due to their low basal expression of GSDME. Moreover, GSDMD cleavage was not observed in any of the cell lines, indicating that chemotherapy-induced pyroptosis proceeds through a GSDME-dependent rather than a GSDMD-mediated pathway (Figure 3a). Cisplatin treatment also increased caspase-3 activation and GSDME cleavage to a greater extent in MKN1-EBV cells than in MKN1 cells, supporting the reproducibility of anticancer drug–induced pyroptotic signaling (Figure S1a,b).

Following PTX treatment, typical pyroptotic features, such as cell swelling and membrane ballooning, were more frequently observed in MKN1-EBV cells than in MKN1 cells (Figure 3b,c). Consistently, LDH release was significantly higher in MKN1-EBV cells (Figure 3d). As both cell lines exhibited resistance to 5-FU, subsequent analyses were performed using PTX only. Collectively, these results indicate that EBV infection enhances chemotherapy-induced GSDME activation and promotes pyroptotic cell death, thereby shifting the cell death pathway from apoptosis to GSDME-mediated pyroptosis.

### 3.4. GSDME Overexpression Shifts PTX-Induced Cell Death Toward Pyroptosis in Gastric Cancer Cells

**Figure 4:**
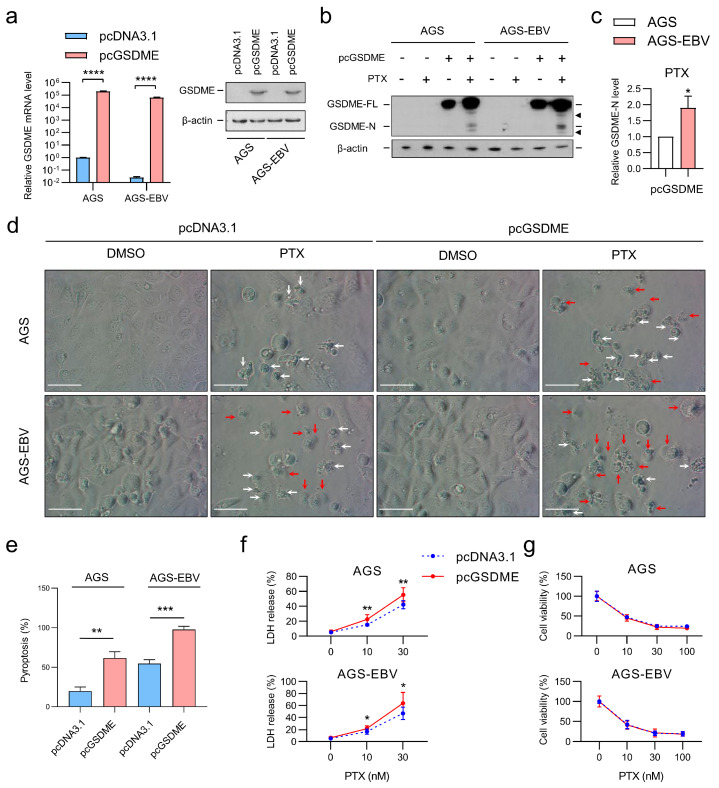
GSDME overexpression enhances PTX-induced pyroptosis in gastric cancer cell lines. (**a**) qRT-PCR (left) and Western blot (right) analyses showing increased *GSDME* expression in AGS and AGS-EBV cells following pc*GSDME* transfection. (**b**–**g**) Cells were transfected with either pcDNA3.1 or pc*GSDME* and subsequently treated with PTX (30 nM) for 48 h. (**b**) Western blot analysis showing GSDME cleavage after PTX treatment. Black triangles indicate non-specific bands. β-actin was used as a loading control. (**c**) Densitometric quantification of cleaved GSDME (GSDME-N) normalized to full-length GSDME (GSDME-FL) in pc*GSDME*-transfected AGS and AGS-EBV cells treated with PTX. Data represent mean ± SD from three independent experiments corresponding to the blots shown in (**b**). (**d**) Representative microscopic images of AGS and AGS-EBV cells treated with PTX for 48 h. Red arrows indicate pyroptotic cells, and white arrows indicate other types of cell death. Scale bar: white horizontal line, 100 µm. (**e**) Quantification of pyroptotic and non-pyroptotic cell death from three independent experiments shown in (**d**). (**f**) LDH release measured in the supernatant of AGS and AGS-EBV cells after PTX treatment. (**g**) Cell viability assessed via MTT assay after PTX treatment. Data are presented as mean ± SD (*n* = 3). Statistical significance is indicated as * *p* < 0.05, ** *p* < 0.01, *** *p* < 0.001, **** *p* < 0.0001.

To further elucidate the role of GSDME in chemotherapy response, AGS and AGS-EBV cells were transfected with either pc*GSDME* or the empty vector pcDNA3.1. qRT-PCR and Western blot analyses confirmed successful overexpression at both the mRNA and protein levels (Figure 4a). PTX treatment enhanced GSDME cleavage in both cell lines, with a more pronounced increase observed in AGS-EBV cells than in AGS cells (Figure 4b,c). Microscopic examination revealed that GSDME overexpression increased the proportion of cells exhibiting pyroptotic morphology in the two cell lines, with the effect being further pronounced following PTX treatment. (Figure 4d,e). Consistently, LDH release was significantly higher in GSDME-overexpressing cells in both AGS and AGS-EBV lines following PTX treatment, indicating membrane rupture and lytic cell death (Figure 4f). Despite the enhanced pyroptotic activity, overall cell viability remained largely unchanged (Figure 4g). These findings corroborate previous reports that GSDME activation converts chemotherapy-induced apoptosis into pyroptosis and further reveal that EBV-infected gastric cancer cells are more susceptible to GSDME-mediated pyroptotic cell death compared with their EBV-negative counterparts.

### 3.5. GSDME Knockdown Suppresses PTX-Induced Pyroptosis

**Figure 5:**
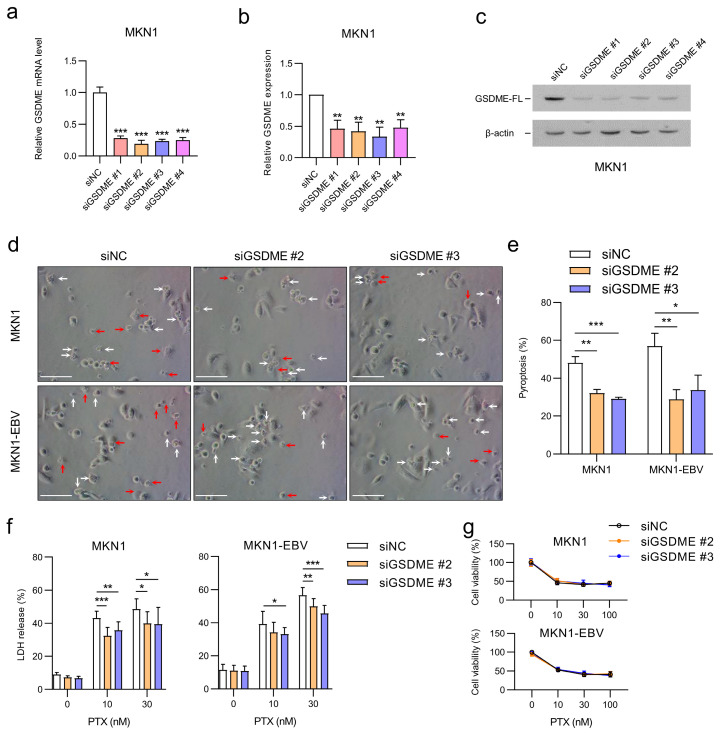
*GSDME*-specific siRNAs suppress PTX-induced pyroptosis in gastric cancer cell lines. (**a**,**b**) MKN1 cells were transfected with four different siRNAs targeting *GSDME* (si*GSDME* #1–4) or a siNC. (**a**) *GSDME* mRNA levels were analyzed via qRT-PCR. (**b**) Expression of GSDME protein was analyzed via Western blotting using three sets of independently prepared cells. The GSDME protein level was normalized to that of β-actin and shown as the ratio compared with the values obtained from the siNC-transfected control cells. (**c**) Representative Western blot analysis of GSDME protein expression as shown in (**b**). β-actin was used as a loading control. (**d**–**g**) MKN1 and MKN1-EBV cells were transfected with the two selected siRNAs (si*GSDME* #2 and #3) or siNC, followed by PTX treatment. (**d**) Representative microscopic images showing the types of cell death. Red arrows indicate pyroptotic cells, and white arrows indicate other types of cell death. Scale bar: white horizontal line, 100 µm. (**e**) Quantification of pyroptotic and non-pyroptotic cell death from three independent experiments shown in (**d**). (**f**) LDH release measured in the supernatant of MKN1 and MKN1-EBV cells after PTX treatment. (**g**) Cell viability assessed via MTT assay after PTX treatment. Data are presented as the mean ± SD (*n* = 3). Statistical significance is indicated as * *p* < 0.05, ** *p* < 0.01, *** *p* < 0.001.

To validate the role of GSDME in PTX-induced pyroptosis, MKN1 and MKN1-EBV cells were transfected with four different siRNAs targeting *GSDME* (si*GSDME* #1–4). All siRNAs effectively reduced *GSDME* mRNA and protein levels compared with the siNC (Figure 5a–c). Based on their efficiency, si*GSDME* #2 and #3 were selected for subsequent experiments. Following PTX treatment, siNC-transfected cells displayed pronounced pyroptotic morphology, whereas GSDME knockdown substantially attenuated these features (Figure 5d,e). Consistently, LDH release was significantly reduced in GSDME-silenced cells (Figure 5d). To further validate membrane permeabilization as an independent indicator of pyroptotic cell death, PI uptake was examined following PTX treatment. The proportion of PI-positive cells was significantly lower in GSDME-knockdown cells than in siNC-transfected control cells, indicating that PTX-induced membrane rupture is GSDME dependent (Figure S2a,b). Despite the reduction in pyroptotic activity, overall cell viability remained largely unaffected (Figure 5g). These results confirm that GSDME is a key mediator of PTX-induced pyroptosis in gastric cancer cells. Silencing GSDME markedly suppresses pyroptotic cell death and shifts cell death signaling toward non-pyroptotic pathways.

### 3.6. Caspase-3 Activity Is Essential for PTX-Induced GSDME-Mediated Pyroptosis

**Figure 6:**
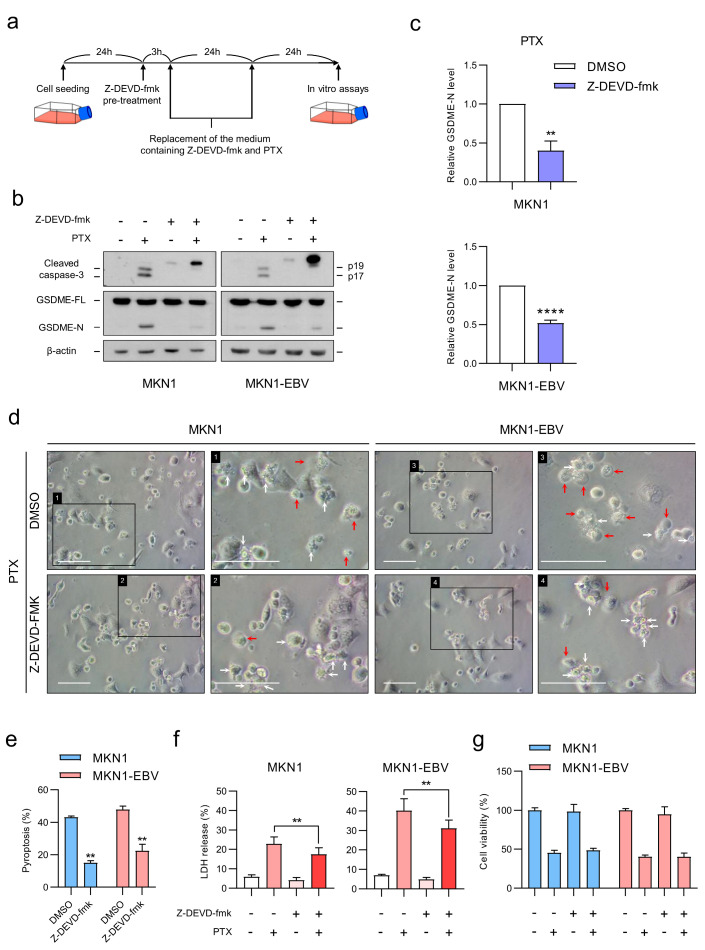
Inhibition of caspase-3 suppresses GSDME-mediated pyroptosis induced by PTX. (**a**) Schematic diagram of the experimental design. Cells were pretreated with Z-DEVD-fmk (20 μM) for 3 h, followed by treatment with fresh medium containing both Z-DEVD-fmk (20 μM) and PTX (30 nM) for 24 h. The medium was then replaced once more with the same drug combination for an additional 24 h before analysis. (**b**) Western blot analysis showing cleavage of caspase-3 and GSDME. β-actin was used as a loading control. (**c**) Densitometric quantification of GSDME-N normalized to GSDME-FL in MKN1 and MKN1-EBV cells treated with PTX in the presence or absence of Z-DEVD-fmk. Quantification is based on three independent experiments corresponding to the blots shown in (**b**). (**d**) Representative microscopic images of cells after co-treatment with Z-DEVD-fmk (20 μM) and PTX (30 nM). Red arrows indicate pyroptotic cells, and white arrows indicate other types of cell death. Scale bar: white horizontal line, 100 µm. (**e**) Quantification of pyroptotic and non-pyroptotic cell death from three independent experiments shown in (**d**). (**f**) LDH release measured in the supernatant of MKN1 and MKN1-EBV cells after PTX treatment. (**g**) Cell viability assessed via MTT assay. Data are presented as the mean ± SD (*n* = 3). Statistical significance is indicated as ** *p* < 0.01, **** *p* < 0.0001.

To determine whether caspase-3 mediates GSDME cleavage upon chemotherapy treatment, MKN1 and MKN1-EBV cells were pretreated with the caspase-3 inhibitor Z-DEVD-fmk (20 μM) for 3 h, followed by co-treatment with PTX and Z-DEVD-fmk for 48 h (Figure 6a). Western blot analysis showed that inhibition of caspase-3 prevented its own activation as well as the subsequent cleavage of GSDME (Figure 6b,c). Consistent with this, Z-DEVD-fmk treatment significantly reduced the proportion of pyroptotic cells and LDH release in both MKN1 and MKN1-EBV cells (Figure 6d–f). However, overall cell viability was not significantly affected (Figure 6g). These results indicate that PTX-induced GSDME cleavage is dependent on caspase-3 activity. Inhibition of caspase-3 suppresses pyroptosis but does not prevent overall cell death, suggesting that blocking caspase-3 shifts the mode of cell death from GSDME-mediated pyroptosis to alternative pathways.

## 4. Discussion

EBV infection contributes to gastric carcinogenesis through extensive epigenetic and transcriptional reprogramming of host cells [38,39]. In this study, we demonstrate that EBV suppresses *GSDME* expression via promoter CpG island hypermethylation and thereby alters the mode of cell death in response to chemotherapy. Analysis of the TCGA dataset revealed that among gasdermin family members, *GSDME* is uniquely downregulated in EBV-positive gastric cancer. Consistently, EBV-infected gastric cancer cell lines exhibited markedly reduced *GSDME* mRNA and protein expression, indicating that EBV selectively targets GSDME to modulate pyroptotic susceptibility.

MSP confirmed hypermethylation of the *GSDME* promoter in EBV-positive and *GSDME* low-expressing cell lines, whereas *GSDME* high-expressing lines showed no such modification. Treatment with the DNA methyltransferase inhibitor 5-AZA reversed this hypermethylation and restored *GSDME* expression. These results align with previous findings that EBV upregulates DNA methyltransferases such as DNMT1, inducing a CpG island methylator phenotype (CIMP) that silences tumor-suppressive genes [38]. Notably, naturally EBV-infected cell lines (SNU719, YCCEL1) reproduced the hypermethylation patterns observed in EBV-positive clinical samples [27], whereas artificially EBV-infected cell lines (AGS-EBV, MKN1-EBV) showed variable *GSDME* promoter methylation, suggesting that EBV-induced epigenetic regulation depends on the preexisting chromatin context of each cell line [40,41,42].

In fact, *GSDME* transcription can also be regulated by p53 binding to an intronic enhancer element located within intron 1 [43]. Therefore, it is possible that EBV may modulate this intronic regulatory site to regulate *GSDME* expression independently of promoter methylation.

In artificially EBV-infected cell lines, *GSDME* expression was decreased despite no detectable increase in promoter methylation. This finding suggests that EBV may employ additional regulatory mechanisms to suppress *GSDME* expression beyond DNA methylation. Notably, *GSDME* transcription can be regulated by p53 through binding to an intronic regulatory element within intron 1 [43]. Therefore, EBV may interfere with this intronic regulatory pathway to inhibit *GSDME* expression independently of promoter methylation. In addition, *GSDME* may be targeted by specific EBV-encoded BART microRNAs, as multiple EBV miRNAs are known to directly bind the 3′ untranslated regions of host genes in EBV-infected gastric cancer cells [10,11]. Further studies are required to determine whether such post-transcriptional regulation of *GSDME* occurs in this context.

Functionally, *GSDME* modulation experiments revealed that this gene dictates the mode rather than the magnitude of cell death. Overexpression of GSDME in low-expressing AGS and AGS-EBV cells markedly enhanced PTX-induced LDH release and pyroptotic morphology, whereas GSDME knockdown in high-expressing MKN1 and MKN1-EBV cells attenuated pyroptotic features without significantly altering overall cell viability. These observations align with previous reports indicating that GSDME mediates a phenotypic shift from apoptosis to pyroptosis without affecting the total extent of cell death, potentially enhancing tumor immunogenicity rather than directly inhibiting cell death [21,22,44].

Although endogenous GSDME expression was very low in both AGS and AGS-EBV cells, chemotherapeutic treatment induced pyroptotic-like morphology even in the absence of exogenous GSDME. While GSDMD activation was not detected under our experimental conditions, other gasdermin family members, such as GSDMA, GSDMB, or GSDMC, may have contributed to plasma membrane damage and lytic cell death. Notably, PTX treatment elicited a stronger pyroptotic response in GSDME-overexpressing AGS-EBV cells than in AGS cells. This difference is likely attributable to higher caspase-3 activity in AGS-EBV cells in response to chemotherapeutic stress, resulting in more efficient cleavage and activation of exogenously expressed GSDME. Importantly, pharmacological inhibition of caspase-3 abolished PTX-induced GSDME cleavage and pyroptotic features, confirming that this process is primarily mediated through the caspase-3-GSDME axis [45,46].

Together, these findings suggest that EBV exerts a dual regulatory effect on pyroptotic signaling: it suppresses endogenous GSDME expression under basal conditions to limit inflammatory cell death, yet enhances caspase-3 activation under chemotherapeutic stress, thereby facilitating rapid execution of pyroptosis once GSDME expression is restored. This dual mechanism may be a way by which the virus balances avoiding immune detection while increasing sensitivity to stress-induced cell death [47,48].

The release of intracellular contents during GSDME-mediated pyroptosis can stimulate local inflammation and enhance anti-tumor immunity by activating macrophages, NK cells, and cytotoxic T lymphocytes [21,49]. Therefore, EBV-driven *GSDME* silencing may contribute to immune evasion in EBVaGC. In contrast, epigenetic reactivation of *GSDME* using demethylating agents such as 5-AZA may restore this pathway. Indeed, studies have shown that 5-AZA pre-treatment enhances GSDME-dependent pyroptosis and promotes dendritic cell maturation and CD8^+^ T-cell infiltration, augmenting the efficacy of nanomedicine-based chemotherapy or gene therapy [50,51].

The increased caspase-3 activity observed in EBV-positive cells may be linked to EBV lytic reactivation. Chemotherapeutic agents such as 5-FU, docetaxel, and 5-AZA are known to induce EBV lytic gene expression, including BZLF1, which is often accompanied by caspase-3 activation [33,52,53,54]. Such lytic reactivation may further facilitate GSDME cleavage and pyroptosis. Future studies are warranted to clarify the interplay between EBV reactivation, caspase-3 activation, and pyroptotic signaling.

In summary, EBV infection epigenetically suppresses *GSDME* expression through promoter hypermethylation, thereby shifting the balance of chemotherapy-induced cell death from pyroptosis to apoptosis. Restoration of GSDME expression reactivates the caspase-3/GSDME pathway, promoting pyroptotic cell death. These findings highlight *GSDME* as a critical mediator of EBV-related epigenetic reprogramming and suggest that combining demethylating agents with chemotherapy may enhance pyroptosis-mediated anti-tumor immunity in EBVaGC. Targeting the EBV–GSDME–caspase-3 axis thus represents a promising therapeutic strategy to overcome immune evasion and improve treatment outcomes in EBVaGC.

## Figures and Tables

**Table 1:** List of primers and their application.

Target	Primer Sequence (5′–3′)	Application
*GAPDH*	F: CATGAGAAGTATGACAACAGCCT	qRT-PCR
	R: AGTCCTTCCACGATACCAAAGT	
*GSDME*	F: GCAAACCACGTGAGTGGAAC	
	R: GTGTCAAAACGCACAGGACC	
*GSDME*	F: CCGGAATTCGCCACCATGTTTGCCAAAGCAACCAG	Cloning
	R: CCGCTCGAGTCATGAATGTTCTCTGCCTA	
Methylated *GSDME* DNA	F: GAGAGTTATACGAAGGAGGGGAAGC	MSP
	R: AAACCTTTACTCGATCCGAACGC	
Unmethylated *GSDME* DNA	F: AGTTATATGAAGGAGGGGAAGTG	
	R: AAACCTTTACTCAATCCAAACAC	

## Data Availability

The original contributions presented in this study are included in the article/Supplementary Materials. Further inquiries can be directed to the corresponding author.

## Supplementary Materials

The following supporting information can be downloaded at https://www.mdpi.com/article/10.3390/microorganisms13122704/s1, Figure S1: Cisplatin induces caspase-3 activation and GSDME cleavage in gastric cancer cells; Figure S2: Propidium iodide (PI) uptake analysis of PTX-induced plasma membrane permeabilization in MKN1 and MKN1-EBV cells.
