# Supplementary material for: Epstein–Barr Virus Silences GSDME and Pyroptosis in Gastric Cancer
Source: Microorganisms. 2025 Nov 27;13(12):2704. doi: 10.3390/microorganisms13122704 (PMC12735423; doi:10.3390/microorganisms13122704)
Supplement: Supplementary file 1 [file microorganisms-13-02704-s001.zip › microorganisms-3971849-supplementary.pdf]

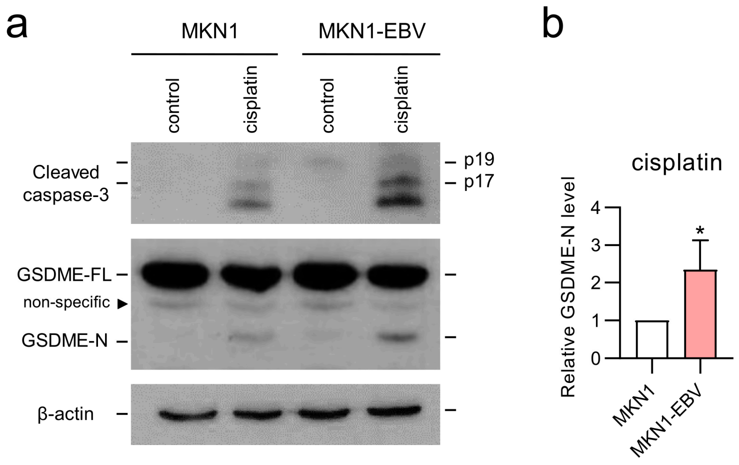

Figure S1. Cisplatin induces caspase-3 activation and GSDME cleavage in gastric cancer cells. (a) MKN1 and MKN1-EBV cells were treated with 10 μM cisplatin or 0.9% NaCl (vehicle control) for 48 hours. Protein levels of caspase-3 and GSDME were analyzed by Western blotting. Black triangles indicate non-specific bands. β-actin was used as a loading control. (b) Densitometric quantification of cleaved GSDME (GSDME-N) normalized to full-length GSDME (GSDME-FL), based on three independent experiments shown in (a).

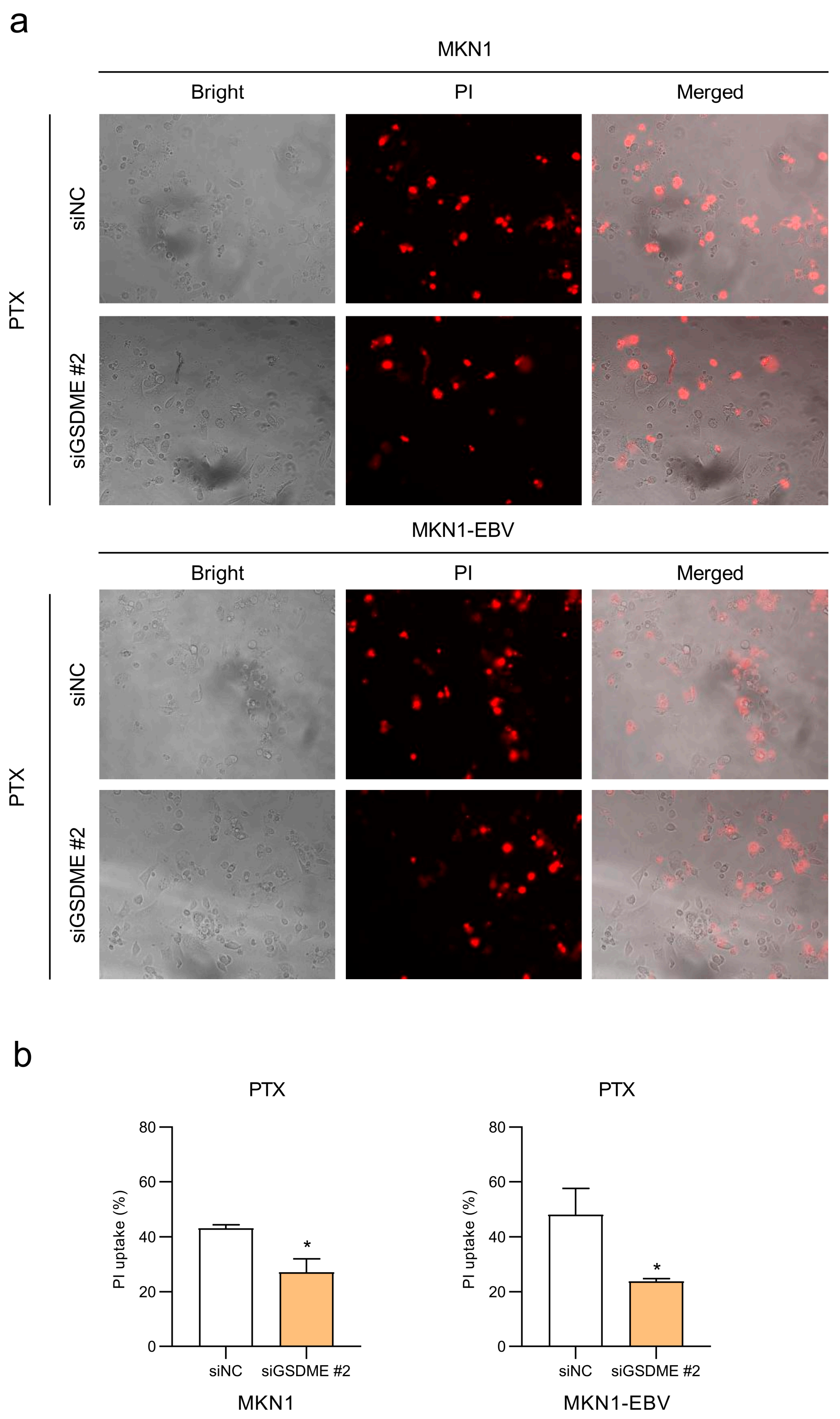

Figure S2. Propidium iodide (PI) uptake analysis of PTX-induced plasma membrane permeabilization in MKN1 and MKN1-EBV cells. (a) Representative fluorescence microscopy images of MKN1 and

MKN1-EBV cells transfected with negative control siRNA (siNC) or siGSDME (#2), followed by treatment with paclitaxel (PTX) for 48 hours. (b) Quantification of PI uptake expressed as the percentage of PI-positive cells, calculated as (number of PI-positive cells / total number of cells) × 100 from the images shown in (a). Data are presented as mean ± SD from three independent experiments ($n = 3$). Statistical significance is indicated as $^*p < 0.05$.
